# Developing Clinical Supervision Guidelines for Nurse Managers to Support Registered Nurses in Lesotho

**DOI:** 10.1002/nop2.71013

**Published:** 2026-11-01

**Authors:** Mpho Zakaria Shelile, Molekodi Jacob Matsipane, Seepaneng Salaminah Moloko‐Phiri, Mmamphamo Miriam Moagi

**Affiliations:** ^1^ Department of Nursing, Faculty of Health Sciences National University of Lesotho Maseru Lesotho; ^2^ School of Nursing Science, Faculty of Health Sciences North‐West University Potchefstroom South Africa; ^3^ Nursing Department, Faculty of Health Sciences University of Limpopo Mankweng South Africa

**Keywords:** AGREE II, clinical supervision, guideline development, healthcare quality, Lesotho, nurse managers, registered nurses

## Abstract

**Aim:**

Clinical supervision supports registered nurses' professional development and ensures quality patient care. In Lesotho, clinical supervision is unstructured, with limited guidance for nurse managers. This study developed evidence‐informed clinical supervision guidelines for nurse managers to support registered nurses in public hospitals in Lesotho and established expert consensus on their content.

**Design:**

A methodological study design.

**Methods:**

A multiphase methodological study design was employed, comprising three phases: Phase 1: concept analysis, Phase 2: empirical exploration with nurse managers and registered nurses and Phase 3: guideline development and expert consensus. Findings from the concept analysis and empirical exploration involving nurse managers and registered nurses were integrated to develop clinical supervision guidelines, guided by a researcher‐developed framework informed by the Appraisal of Guidelines for Research and Evaluation II instrument and Donabedian's model. A three‐round Electronic Delphi survey with 21 national and international experts was conducted to establish consensus on the validity, reliability, applicability, clarity, relevance, comprehensiveness, flexibility and acceptability of the guidelines.

**Results:**

Eight context‐specific clinical supervision guidelines were developed, organised into structure and process domains to ensure an outcome of comprehensive and effective clinical supervision. Guideline 8 was classified as structural, guidelines 1, 2, 3, 4, 5 and 7 were classified as process‐oriented and guideline 6 was considered to have both structural and process characteristics. Expert consensus was achieved on all guidelines.

**Conclusion:**

The clinical supervision guidelines, developed through expert consensus, provide a structured, evidence‐informed and consensus‐based framework that can strengthen nurse managers' clinical supervision, support and mentorship of registered nurses, while supporting registered nurses' competence and well‐being, with potential benefits for patient‐care quality. Adoption of these guidelines could enhance an efficient, supportive and accountable nursing care environment in Lesotho.

**Implications for Nursing Practice:**

The clinical supervision guidelines may provide a structured, evidence‐informed and consensus‐based framework for standardising nurse managers' clinical supervision in public hospitals. Their implementation could strengthen accountability, communication, professional support, workforce development and performance management, while improving registered nurses' competence and overall healthcare quality.

**Impact:**

The study provides an evidence‐informed, context‐specific framework to standardise clinical supervision, strengthen nurse managers' supervisory capacity, improve registered nurses' competence and well‐being and enhance patient safety and the overall quality of healthcare in Lesotho's public hospitals.

**Reporting Method:**

This multiphase study was reported in accordance with four reporting methods, namely: The Quality Appraisal Tool for Studies with Diverse Designs, Standards for Reporting Qualitative Research, Accurate Consensus Reporting Document and Reporting Items for Practice Guidelines in Healthcare.

**Patient or Public Contribution:**

This study did not include patient or public involvement in its design, conduct, or reporting.

## 1 Background

Guidelines are systematically developed recommendations aimed at optimising patient care, informed by evidence and a balanced evaluation of the benefits and risks associated with different care options (Shekelle et al. 2018). Diverse stakeholders, including hospitals, government agencies, professional organisations, or international bodies, may develop guidelines. Variations in guidelines often stem from differences in professional backgrounds, operational systems, resource availability, methodological approaches and cultural or contextual factors (Salarvand et al. 2020). For guidelines to effectively influence patient care, they must be carefully developed and evaluated prior to dissemination to healthcare providers and implementation in routine practice. The development of rigorous, evidence‐informed guidelines is often one of the more straightforward stages, as demonstrated by numerous agencies (Cassidy et al. 2021). To support informed decision‐making, tools are now available to evaluate the quality and relevance of clinical guidelines. For instance, the Appraisal of Guidelines for Research and Evaluation (AGREE) Collaboration focuses on assessing guideline content and has developed a tool that enables users to appraise the quality of recommendations within guidelines (AGREE II 2017). Healthcare institutions may therefore benefit from adopting guidelines that have already been tested and validated, allowing them to concentrate their efforts and resources on local implementation; an undertaking that is not always straightforward (Qaseem et al. 2012). In the context of clinical supervision in nursing practice, evidence‐informed guidelines are essential for standardising supervisory practices, ensuring consistent support for registered nurses and improving overall quality of care (Rosenfeld et al. 2013). Clearly defined clinical supervision guidelines in nursing practice provide nurse managers with a structured framework for supervision by clarifying roles, responsibilities and best practices in mentoring and professional development (Cassidy et al. 2021). The development of such guidelines is particularly crucial in strengthening workforce capacity and improving patient outcomes, especially in settings like public hospitals in Lesotho, where effective clinical supervision in nursing practice can significantly impact nursing performance and healthcare delivery (Ernawati et al. 2022). By establishing clear, actionable guidelines, healthcare institutions can ensure a supportive environment for registered nurses, enabling nurse managers to deliver structured, high‐quality supervision that aligns with professional standards and organisational goals (De Leo et al. 2023).

This paper reports on the development of clinical supervision guidelines in nursing practice and presents these guidelines as part of a broader project. In the first phase of this study, a concept analysis established that clinical supervision in nursing practice is a structured and supportive professional development process in which registered nurses reflect on their practice within an environment of confidentiality, safety and trust, guided by a competent nurse manager. This process is initiated by the healthcare institution's commitment to delivering quality nursing care, supported by professional expectations and demands and underpinned by the acquisition and application of relevant knowledge and skills in collaboration with community members and partners (Ryu et al. 2024). Clinical supervision in nursing practice is distinct from resource management, which concerns the allocation and utilisation of human, material and financial resources; leadership, which focuses on influencing and directing staff towards shared organisational and clinical goals; and administrative supervision, which addresses organisational policies, procedures, staffing and accountability (Valimaki et al. 2024; WHO 2022). Quality of care refers to the extent to which health services are safe, effective, timely, people‐centred and responsive to patients' needs, while performance evaluation involves the systematic assessment of individual staff performance against defined standards. Although these domains are interrelated, clinical supervision in nursing practice primarily focuses on supporting and improving clinical practice and professional development, while the other domains provide complementary managerial and organisational functions (Valimaki et al. 2024; WHO 2022). Effective clinical supervision in nursing practice can lead to improved performance, increased job satisfaction and enhanced quality of nursing care. According to Saab et al. (2021), clinical supervision in nursing practice promotes alignment with organisational goals, standards and professional development while strengthening registered nurses' knowledge and decision‐making skills. However, challenges such as low engagement and disruptions related to workload remain (Yuswanto et al. 2018). Further to that, clinical supervision in nursing practice provides registered nurses with opportunities for professional reflection, contributing to improved care documentation, increased confidence and enhanced overall well‐being (Saab et al. 2021).

Hancock and Meadows (2020) assert that effective nurse managers ensure a supervisory partnership built on mutual trust and respect. The authors also state that nurse managers act as role models, safeguard public safety and uphold professional standards by ensuring supervisees meet competency requirements. Similarly, Pinto et al. (2017) recommended that in Portugal, clinical supervision in nursing practice should incorporate observation, demonstration, feedback and support to encourage reflective practice, thereby enhancing knowledge, skills and professional growth. Falender et al. (2014) further indicates the importance of structured guidelines and standards in clinical supervision to promote their acceptance and effectiveness.

However, evidence from the literature suggests that the effective implementation of clinical supervision for registered nurses remains insufficiently defined. While in the UK there is the NHS England Advocating and Educating for Quality Improvement (A‐EQUIP) model of professional nursing leadership and clinical supervision (NHS England 2023), Pollock et al. (2017) identified a notable gap in empirical evidence from some developed countries regarding optimal approaches to implementing clinical supervision in nursing practice, largely due to the absence of standardised guidelines. This observation is reinforced by Rothwell et al. (2019), who report limited evidence concerning the appropriate frequency and duration of clinical supervision in nursing practice. These gaps highlight the need for structured, context‐sensitive clinical supervision frameworks that can guide consistent practice. In this regard, Lee et al. (2020) contend that clinical supervision in developed settings should focus on strengthening ongoing nursing processes and ensuring that registered nurses perform their roles with maximum efficiency, a view that underscores the importance of evidence‐informed and operationally focused supervision models.

Evidence from African contexts indicates that clinical supervision in nursing practice plays a meaningful role in strengthening relationships among registered nurses, nurse managers and other healthcare professionals, with positive implications for patient care (Alhassan et al. 2025). However, a closer examination of the literature suggests that these benefits are often realised alongside persistent systemic and leadership‐related challenges. Studies conducted across the continent revealed both the potential and the limitations of clinical supervision in nursing and midwifery practice. For example, in Ghana, Achempim‐Ansong et al. (2022) demonstrated that effective clinical supervision in nursing practice was associated with reduced infection rates, lower levels of negligence and increased professional recognition among registered nurses. These outcomes stress the value of structured supervision in improving care quality. Nonetheless, the sustainability of such gains remains questionable where leadership capacity is insufficient. Adatara et al. (2018) reported that nurse managers in Ghana's Volta Region frequently experience stress stemming from inadequate preparation for managerial roles, unclear job descriptions and limited management skills, factors that significantly constrain the effective implementation of clinical supervision in nursing practice.

Similar patterns are evident across several African countries, suggesting that the effectiveness of clinical supervision in nursing practice is closely linked to both supervisory capacity and contextual support. In Egypt, ElZeneny et al. (2017) demonstrated that a clinical supervision training programme significantly enhanced nurse managers' supervisory skills, with improvements sustained 3 months post‐training. This finding highlights the value of targeted capacity‐building interventions. Mohamed and Ahmed (2019) further emphasised that a supportive ward environment is critical to maximising the impact of clinical supervision in nursing practice. Serapelwane et al. (2025) reported that, in sub‐Saharan Africa, clinical supervision in nursing practice improved registered nurses' confidence and reduced stress levels, while in Tanzania, Purity et al. (2017) stressed the importance of structured guidelines in facilitating effective supervisory interactions. From the author's perspective, these findings collectively point to the necessity of context‐appropriate and well‐structured supervision frameworks with adequate clinical governance systems. However, the literature also reveals important limitations. Gundani (2018) observed that nurse managers in Zimbabwe perceived checklists as cumbersome and redundant for routine clinical supervision in nursing practice, indicating that overly rigid tools may hinder rather than support practice. Furthermore, resource limitations and psychological readiness of nurse managers continue to impede effective supervision. In South Africa, Raliphaswa et al. (2021) identified shortages of resources and inadequate mental preparedness as significant barriers to supervision, while in Botswana, Boitshwarelo et al. (2020) noted that registered nurses' expectations for managerial support, particularly in complex clinical situations, highlight the ongoing need for skilled and responsive clinical supervision.

Lesotho is a small, mountainous, landlocked country in Southern Africa with a predominantly rural population and a healthcare system comprising public, private and non‐governmental health facilities. The Ministry of Health is responsible for the public healthcare system, including policy development, service delivery, human resources and regulation. Healthcare services are organised across primary, secondary and tertiary levels, with primary healthcare facilities serving as the first point of contact and hospitals providing referral and specialised services. Hospitals include district, regional and national referral facilities and provide medical, surgical, maternity, paediatric, emergency and specialist services. Registered nurses constitute a substantial proportion of the healthcare workforce and play a central role in the delivery and coordination of patient care. Nursing education is provided through accredited institutions and combines theoretical and clinical learning, with hospitals serving as important clinical learning environments for developing and maintaining professional competence.

Within hospital settings, nurse managers are responsible for coordinating nursing services, allocating resources, monitoring nursing practice, facilitating professional development and promoting the quality and safety of patient care. Nurse managers provide clinical supervision to registered nurses as part of their managerial and professional responsibilities, providing guidance, feedback, professional support and opportunities for learning and reflection while monitoring adherence to professional standards and organisational requirements. However, in Lesotho, clinical supervision in nursing practice remains largely unstructured and lacks a clear, shared definition. Although initiatives such as the Nursing Education Partnership Initiative (NEPI) offered short‐term support to newly recruited registered nurses, these interventions were not sustainable and did not adequately include experienced practitioners (Middleton et al. 2014). A key lesson from this initiative was that strengthening nursing practice requires interventions that extend beyond initial orientation or recruitment and that recognise the ongoing developmental needs of experienced registered nurses. The limitations also highlighted the importance of considering contextual barriers, including limited human and material resources, workload pressures, inadequate opportunities for continuing professional development and variations in managerial capacity to provide ongoing clinical supervision in nursing practice. These lessons informed the design and conduct of the present study by recognising clinical supervision in nursing practice as a continuous process that should support registered nurses at different stages of their careers rather than focusing primarily on newly recruited registered nurses. The absence of formal guidelines for clinical supervision in nursing practice in Lesotho has contributed to fragmented and inconsistent supervisory practices. Without clear guidance to direct nurse managers, clinical supervision is often implemented in an uncoordinated manner, which limits the effective professional support provided to registered nurses. This lack of clinical supervision guidelines leads to unstructured supervision, which contributes to reduced motivation among registered nurses and may negatively affect the quality and safety of patient care (Government of Lesotho 2013). In response to these challenges, this study aimed to develop and evaluate evidence‐informed and consensus‐based clinical supervision guidelines to address the identified gaps and strengthen supervisory practice within the Lesotho healthcare context.

## 2 Methods

### 2.1 Aim

Given the identified gaps, including the absence of formal clinical supervision guidelines and unstructured clinical supervision in Lesotho, this study aimed to develop evidence‐informed and consensus‐based clinical supervision guidelines for nurse managers to support registered nurses in public hospitals in Lesotho.

### 2.2 Design

A multiphase methodological study design was used, conducted in the following three phases: concept analysis, empirical phase and development and evaluation through expert consensus. Although the guideline development process was informed by findings from preceding concept analysis and qualitative studies, the present paper provides sufficient methodological detail to enable readers to understand and assess how the clinical supervision guidelines were developed, evaluated and refined through expert consensus. This multiphase study was reported in accordance with four reporting methods, namely: The Quality Appraisal Tool for Studies with Diverse Designs, Standards for Reporting Qualitative Research, Accurate Consensus Reporting Document and Reporting Items for Practice Guidelines in Healthcare.

### 2.3 Setting of the Study

The study was conducted by the researcher at public hospitals in Lesotho, located in the districts of Mohale's Hoek, Mafeteng, Maseru, Berea, Leribe and Butha‐Buthe. These districts were selected because they span from lowland to mountainous regions, encompassing rural areas.

The selected hospitals represented diverse geographical and service‐delivery contexts, including urban, peri‐urban, rural, lowland and mountainous settings. This diversity was considered important for capturing variation in the contexts in which nurse managers provide clinical supervision to registered nurses and for ensuring that the development of the clinical supervision guidelines was informed by experiences across different public hospital settings in Lesotho.

### 2.4 Phase 1: Concept Analysis

The aim of the concept analysis was to clarify the meaning of clinical supervision in the context of nursing practice. The model developed by Walker and Avant (2014) was chosen for this analysis due to its clarity, systematic approach and suitability for use by novice researchers or those conducting concept analysis. The detailed description of the eight steps, namely, (1) Selection of a concept for analysis, (2) Determining the aims and purposes, (3) Identifying all uses of the concept, (4) Determining the defining attributes, (5) Identifying a model case, (6) Identifying additional cases, (7) Identifying antecedents and consequences and (8) Defining empirical referents as proposed by Walker and Avant (2014) were followed. Data were sourced from databases such as Google Scholar, CINAHL, African Journals, ScienceDirect and Health; Source: Nursing/Academic edition. The researcher searched titles and abstracts of articles published between 2003 and 2023 in January and February 2025, using keywords such as ‘clinical nursing supervision’, ‘clinical’, ‘supervision’, ‘clinical supervision’ and ‘clinical supervision in nursing’. A total of 435 articles and sources were identified, of which 71 (*N* = 71) definitions and uses of clinical supervision were recorded from the literature and used in the analysis. The Preferred Reporting Items for Systematic Reviews and Meta‐Analysis (PRISMA) flow diagram was used to eliminate studies which were somewhat irrelevant, keeping the most relevant studies for inclusion in this concept analysis. The Quality Appraisal Tool for Studies with Diverse Designs (QATSDD), proposed by Sirriyeh et al. (2012), was used to assess the quality of the studies included in this concept analysis. The theoretical sample was determined by data saturation, meaning the search continued until no new definitions or uses of the concept of clinical supervision in nursing were found in the literature. The findings from concept analysis revealed that competency, ability to reflect and a safe environment are antecedents for clinical supervision in nursing practice. The process of clinical supervision includes the establishment of a relationship, reflection and professional development. The outcomes of clinical supervision in nursing practice are improved performance, increased job satisfaction and better quality of nursing care. Based on the findings of the concept analysis, the authors proposed the following denotative definition of clinical supervision: Clinical supervision, in nursing practice, is a structured, supportive professional development process in which registered nurses reflect on their practice within the context of confidentiality, safety, trust and with a competent nurse manager.

### 2.5 Phase 2: Empirical Phase

This phase consisted of two steps where qualitative data were collected from nurse managers and registered nurses and analysed independently to inform the development of clinical supervision guidelines. Participants were purposively recruited from selected public hospitals in Lesotho to ensure the inclusion of nurse managers and registered nurses with relevant clinical experience of clinical supervision. Nurse managers participated in face‐to‐face interviews, while registered nurses participated in focus group discussions (FGDs). Participation in both data collection activities was voluntary, and participants were informed of their right to decline participation or withdraw from the study at any time without penalty or adverse consequences. To minimise potential selection and researcher bias, clearly defined inclusion and exclusion criteria were applied consistently across all participating hospitals. Where feasible, recruitment was undertaken without the involvement of participants' direct supervisors to minimise the possibility of perceived coercion and support voluntary participation. The researcher maintained reflexivity throughout data collection and analysis by keeping field notes and documenting methodological decisions. Confidentiality and anonymity were maintained throughout the empirical phase. Unique identification codes were assigned to participants, and their names were not used in transcripts or the reporting of findings. For the FGDs, participants were also informed of the importance of respecting the confidentiality of information shared during discussions, although confidentiality among group members could not be guaranteed by the researcher. Ethical clearance for the study was obtained from the North‐West University Health Research Ethics Committee (NWU‐00175‐23‐A1), and permission to conduct the study in Lesotho was granted by the Ministry of Health (ID20‐2024). Both approvals covered all phases of the study. The trustworthiness of the study was ensured by adhering to the four established criteria for assessing qualitative research trustworthiness: credibility, dependability, confirmability and transferability (Johnson et al. 2020). These criteria guided the conduct of both the face‐to‐face interviews with nurse managers and the focus group discussions with registered nurses. Credibility was enhanced through reflexivity, iterative analysis, peer debriefing and independent verification of codes and categories. Throughout the research process, the researcher engaged in continuous critical reflection on how his professional background and experience might influence interactions with participants and the interpretation of the data. Both the face‐to‐face interviews and FGDs were audio‐recorded, and field notes were taken to document contextual information and relevant non‐verbal cues. Open‐ended questions and probing were used to encourage participants to elaborate on their responses and clarify their views. The researcher and co‐coder independently read and coded the transcripts several times before engaging in peer debriefing to compare and refine their interpretations. Discrepancies were discussed until consensus was reached, helping to ensure that the categories and themes reflected participants' accounts and minimised potential researcher bias. Following data analysis, participants were consulted to validate the findings (Johnson et al. 2020). Dependability was ensured by maintaining a transparent and systematic record of the research process, including data collection, transcription, coding and categorisation. An audit trail documented methodological decisions and the development and refinement of categories and themes across the interview and focus group data. Detailed transcription, systematic coding and analysis and consultation of relevant literature further strengthened dependability (Johnson et al. 2020). These procedures provide a clear account of the analytical process and enable readers to follow how the findings were developed. Confirmability was strengthened through independent analysis of the interview and focus group transcripts by the researcher and co‐coder. After completing their initial analyses, they compared the emerging codes, categories, themes and sub‐themes, discussed differences in interpretation and reached consensus. The use of open‐ended questions and probing during both data collection methods helped ensure that the findings were grounded in participants' accounts. Relevant literature was subsequently consulted to support, contextualise and critically examine the findings (Johnson et al. 2020). These strategies helped minimise the influence of the researcher's assumptions and ensured that the interpretations were supported by the data. Transferability was supported by providing a detailed description of the study setting, participant characteristics, data collection methods and analytical procedures. Reporting the contextual differences between the face‐to‐face interviews with nurse managers and the FGDs with registered nurses also enables readers to understand the perspectives represented in the findings. This detailed contextual description allows readers to assess the extent to which the findings may be applicable to similar settings or populations (Johnson et al. 2020). The two steps are briefly described below.

#### 2.5.1 Step 1: Qualitative Data From Nurse Managers

This step of the study employed an exploratory descriptive qualitative research design to explore and describe the support provided to registered nurses during clinical supervision. Data were gathered through eight individual face to face interviews with participants purposefully selected from the public hospitals in Lesotho. The face to face interviews lasted for 45–60 min and were recorded and transcribed verbatim for analysis. The researcher initially planned to conduct at least 12 interviews; however, no new codes or themes emerged by the seventh interview. An eighth interview was conducted to assess whether saturation had been reached. Data were analysed by the researcher and the co‐coder using thematic analysis as proposed by Braun and Clarke (2013). Three themes emerged from the data: nurse managers' supervisory dimensions to facilitate healthcare quality, nurse managers' supervisory practices to support registered nurses and nurse managers' challenges with clinical supervision. Providing structural support to registered nurses, applying methods and processes to supervise registered nurses and supervising registered nurses to enhance patient outcomes is the fundamental support given to registered nurses by nurse managers during clinical supervision. Nurse managers guide registered nurses through performance monitoring, quality assurance and resource supervision to uphold standards and policies. Supervision of registered nurses also support skill development, conflict management and psychosocial well‐being. However, challenges include addressing registered nurses' attitudes, managing skill gaps and overcoming nurse managers' own knowledge limitations. The following conclusions were drawn from the themes: Conclusive statement, Theme 2.1.1: Effective supervision promotes accountability and quality of care through performance monitoring, quality assurance and supervision of human and material resources. Conclusive statement, Theme 2.1.2: Clinical supervision supports knowledge and skill development and ensures that registered nurses have the competencies required for patient care. Conclusive statement, Theme 2.1.3: Nurse managers encounter difficulties supervising registered nurses with knowledge and skills gaps and acknowledge limitations in their own supervisory knowledge and capacity.

#### 2.5.2 Step 2: Qualitative Data From Registered Nurses

This step of Phase 2 of the study explored the experiences of registered nurses during clinical supervision with nurse managers in public hospitals in Lesotho. A qualitative descriptive design was adopted using focus group discussions. The focus group discussions (FGDs) were facilitated by the researcher, who had formal training in qualitative research and experience in nursing, healthcare management and nursing education. The facilitator had no hierarchical or supervisory relationship with the participants, thereby minimising potential power imbalances and reducing the risk of response bias. A total of 60 registered nurses participated in eight FGDs, with each group comprising 6–8 participants. The FGDs, which lasted for 45–60 min, were conducted in a conducive setting and were audio‐recorded with participants' consent to ensure accurate capture of the discussions. An independent person was present during each FGD to take field notes, including observations of non‐verbal reactions, interruptions and other contextual factors that could contribute to interpretation of the data. Data saturation was reached by the 6th focus group discussion, with two additional focus group discussions conducted to confirm this. Data were analysed by the researcher and an independent co‐coder using thematic analysis as proposed by Braun and Clarke (2013). Four themes emerged from the data; namely: challenges experienced by registered nurses in hospitals during clinical supervision, inadequate methods and processes of maintaining quality patient care in hospitals, inadequate supportive supervision of registered nurses and unclear supervisory role by nurse managers. Registered nurses said there are challenges that can hinder effective nursing practice and overall care delivery, particularly in hospitals in Lesotho, where the support and clinical supervision provided are crucial for ensuring high‐quality patient care. While clinical supervision has the potential to significantly enhance patient care, staff development and workplace culture, its effectiveness is currently hindered by multiple systemic challenges. These include chronic staff shortages, inadequate resource allocation, ineffective communication, poor access to updated guidelines, lack of structured performance evaluation and inconsistent supervisory practices. The following conclusive statement emerged from the themes: Conclusive statement, Theme 2.2.1: Human and material resource shortages and limited access to policies, guidelines and protocols compromise supervision. Conclusive statement, Theme 2.2.2: Inadequate leadership and quality assurance monitoring weaken accountability and standards of care. Conclusive statement, Theme 2.2.3: Fair and effective conflict management and staff well‐being support, including counselling and wellness programmes, are needed to promote a positive workplace culture and manage work‐related stress. Conclusive statement, Theme 2.2.4: Nurse managers need to orient new employees, clarify expectations, strengthen supervisory knowledge and skills and establish clear supervisory structures and roles.

### 2.6 Phase 3: Development and Evaluation Phase

The results of the first two phases were used to develop the proposed clinical supervision guidelines. The Donabedian (2003) model provided a conceptual basis for organising the content of the guidelines around the structural conditions required for effective clinical supervision, the processes through which supervision is conducted and the outcomes expected to improve nursing practice and healthcare quality. The AGREE II (2017) Instrument was used to develop a methodological framework that guided the development of the clinical supervision guidelines. The AGREE II was therefore used to inform the development framework rather than as a standalone guideline development methodology.

#### 2.6.1 Step 1: Development of Clinical Supervision Guidelines

A researcher‐developed methodological framework guided the development of context‐specific clinical supervision guidelines for nurse managers supporting registered nurses in public hospitals in Lesotho, informed by the six domains and 23 items of the AGREE II instrument (Figure 1). The AGREE II domains and items informed the development process rather than being treated as 23 independent clinical supervision guidelines development frameworks. Their application supported consideration of the guidelines' methodological quality and implementation requirements.

**FIGURE 1:**
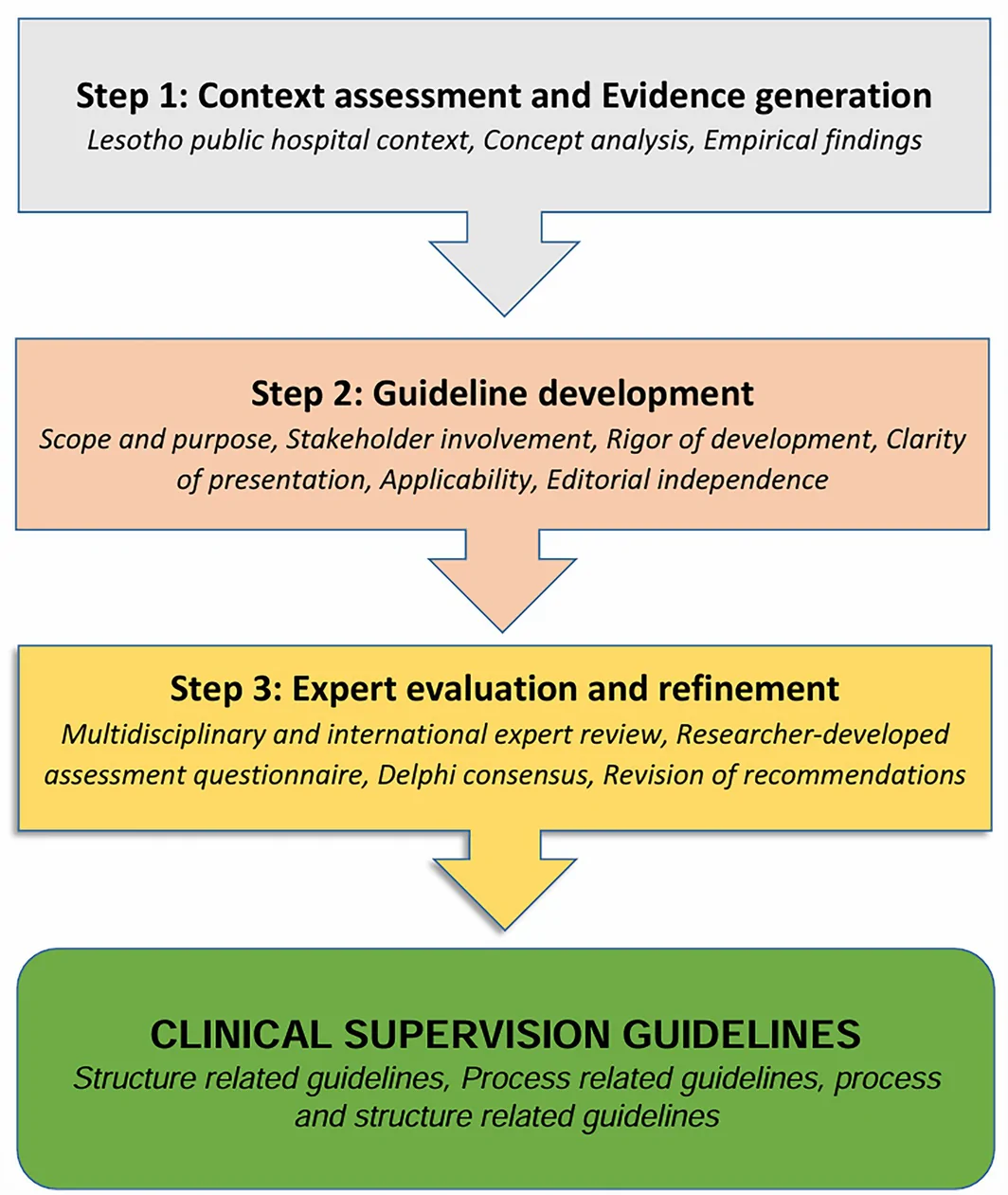
Guideline development framework.

##### 2.6.1.1 Step 1.1 Context Assessment and Evidence Generation

The framework integrated findings from concept analysis and empirical investigations of clinical nursing supervision in Lesotho's public hospitals (Figure 2). Phase 1, the concept analysis, identified the key attributes, antecedents and consequences of clinical supervision, providing the conceptual foundation for the guidelines. Phase 2 generated empirical findings on supervisory practices, contextual challenges and the support needs of nurse managers and registered nurses in clinical settings. These findings were synthesised into seven conclusive statements. The conceptual insights and empirical findings were subsequently integrated to formulate the guidelines. The researcher systematically mapped the conceptual attributes, processes and anticipated outcomes against the seven conclusive statements to identify areas of convergence, complementary evidence and gaps requiring consideration during guideline formulation. Each guideline was developed to address an identified supervisory function, an empirically established support need or challenge, or a theoretically grounded component of effective clinical supervision. For example, the findings concerning performance monitoring and inadequate quality assurance informed the guideline on direct clinical supervision, while evidence of knowledge and skills gaps and inadequate staff orientation informed the guideline on clinical learning and professional development. Similarly, the conceptual importance of reflection informed the guideline on reflective clinical practice, while the qualitative findings concerning limited managerial engagement, inadequate supervisory structures and insufficient follow‐up informed the guideline on structured and continuous supervision (Table 1). Where conceptual and empirical findings converged, their alignment strengthened the evidential justification for the relevant guidelines. Where guidelines were primarily grounded in the concept analysis, as in the emphasis on reflection, their conceptual basis was retained without implying direct empirical confirmation.

**FIGURE 2:**
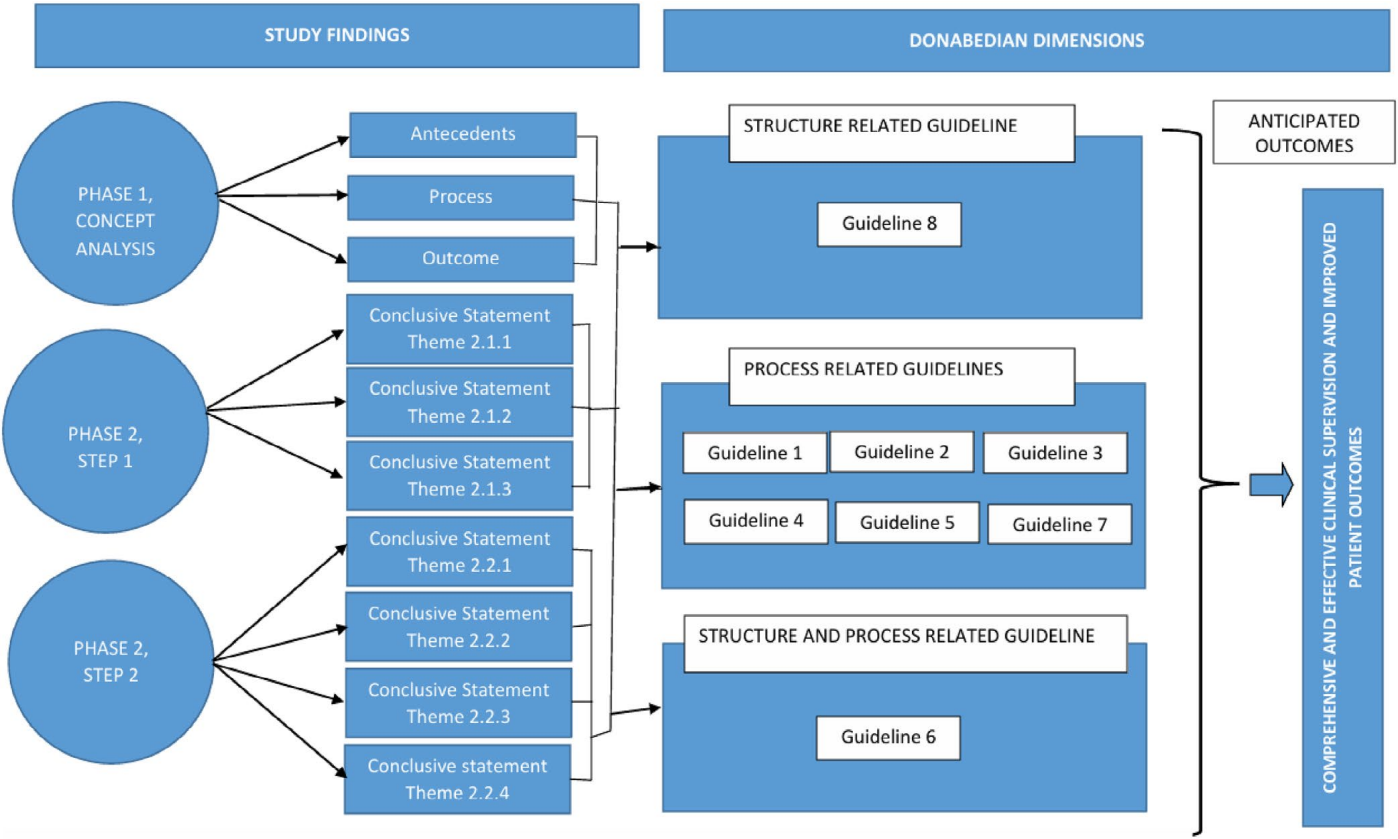
Framework of the integrating study findings in the development of clinical supervision guidelines.

**TABLE 1:** Guideline linkage to the concept analysis and qualitative findings.

Guidelines	Findings from concept analysis	Conclusive statements from empirical phase	Evidence‐to‐guideline rationale
1. Conduct direct clinical supervision to support registered nurses in the safe and effective delivery of patient care.	A safe environment and nurse manager competency are antecedents of clinical supervision. Improved performance and better quality of nursing care are expected outcomes.	Conclusive statement, Theme 2.1.1: Effective supervision promotes accountability and quality of care through performance monitoring, quality assurance and supervision of human and material resources. Conclusive statement, Theme 2.2.1: Human and material resource shortages and limited access to policies, guidelines and protocols compromise supervision. Conclusive statement, Theme 2.2.2: Inadequate leadership and quality assurance monitoring weaken accountability and standards of care.	The convergence of conceptual and empirical evidence supports direct supervision as a mechanism for monitoring clinical practice, reinforcing adherence to nursing standards, identifying unsafe practices and promoting patient safety. Implementation requires attention to resource availability and access to clinical guidance.
2. Facilitate clinical learning and professional development through supportive supervision.	Professional development is a core process of clinical supervision. Nurse manager competency is an antecedent. Improved performance and job satisfaction are expected outcomes.	Conclusive statement, Theme 2.1.2: Clinical supervision supports knowledge and skill development and ensures that registered nurses have the competencies required for patient care. Conclusive statement, Theme 2.1.3: Nurse managers encounter difficulties supervising registered nurses with knowledge and skills gaps and acknowledge limitations in their own supervisory knowledge and capacity. Conclusive statement, Theme 2.2.4: Nurse managers need to orient new employees, clarify expectations, strengthen supervisory knowledge and skills and establish clear supervisory structures and roles.	These findings support supervision as a continuous learning process that strengthens clinical competence. The guideline therefore emphasises identifying learning needs, facilitating continuing professional development, supporting newly appointed staff and strengthening nurse managers' supervisory competence.
3. Promote reflective clinical practice and evidence‐informed decision‐making among registered nurses.	The ability to reflect is an antecedent of clinical supervision Reflection is a core supervisory process. Professional development and improved performance are expected outcomes.	Conclusive statement, Theme 2.1.1: Effective supervision promotes accountability and quality of care through performance monitoring, quality assurance and supervision of human and material resources. Conclusive statement, Theme 2.2.1: Human and material resource shortages and limited access to policies, guidelines and protocols compromise supervision. Conclusive statement, Theme 2.1.2: Clinical supervision supports knowledge and skill development and ensures that registered nurses have the competencies required for patient care.	The concept analysis directly establishes reflection as integral to supervision. The qualitative findings support the need to strengthen clinical knowledge and access to evidence‐informed guidance. Reflective practice is therefore recommended to help registered nurses critically examine clinical decisions, identify learning needs and apply available evidence to patient care. Reflection itself was not explicitly identified in the supplied qualitative conclusive statements and should be reported as primarily conceptually grounded.
4. Provide constructive clinical feedback to strengthen nursing practice and patient safety.	The establishment of a supervisory relationship and the creation of a safe environment support clinical supervision. Improved performance and better quality of nursing care are expected outcomes.	Conclusive statement, Theme 2.1.1: Effective supervision promotes accountability and quality of care through performance monitoring, quality assurance and supervision of human and material resources. Conclusive statement, Theme 2.2.2: Inadequate leadership and quality assurance monitoring weaken accountability and standards of care. Conclusive statement, Theme 2.1.3: Nurse managers encounter difficulties supervising registered nurses with knowledge and skills gaps and acknowledge limitations in their own supervisory knowledge and capacity.	The evidence supports constructive feedback as a means of communicating performance expectations, reinforcing good practice, identifying deficiencies and guiding corrective action. Feedback should be specific, respectful, timely and directed towards professional development and improved patient safety. The explicit formulation of constructive feedback is a guideline derived from the broader evidence on monitoring, performance improvement and corrective supervision.
5. Facilitate clinical problem‐solving and management of complex patient‐care situations.	Competency and the ability to reflect are antecedents that support effective supervision. Reflection and professional development are processes through which clinical competence and performance may improve.	Conclusive statement, Theme 2.1.2: Clinical supervision supports knowledge and skill development and ensures that registered nurses have the competencies required for patient care. Conclusive statement, Theme 2.1.3: Nurse managers encounter difficulties supervising registered nurses with knowledge and skills gaps and acknowledge limitations in their own supervisory knowledge and capacity. Conclusive statement, Theme 2.1.1: Effective supervision promotes accountability and quality of care through performance monitoring, quality assurance and supervision of human and material resources.	These findings support the need for nurse managers to guide clinical reasoning, facilitate collaborative problem‐solving, identify appropriate interventions and support registered nurses in managing difficult clinical situations. The guideline also highlights the need to strengthen nurse managers' own clinical and supervisory competencies.
6. Promote supportive interpersonal relationships that enable registered nurses to discuss clinical and professional concerns.	Establishing a relationship and creating a safe environment are central to clinical supervision. The process includes reflection and professional development, with increased job satisfaction as an expected outcome.	Conclusive statement, Theme 2.1.2: Clinical supervision supports knowledge and skill development and ensures that registered nurses have the competencies required for patient care. Conclusive statement, Theme 2.2.3: Fair and effective conflict management and staff well‐being support, including counselling and wellness programmes, are needed to promote a positive workplace culture and manage work‐related stress.	The convergence of findings supports a trusting and supportive supervisory relationship in which registered nurses can raise concerns, discuss clinical uncertainties, reflect on practice and seek assistance without fear of humiliation or unfair treatment. Such relationships can also support professional development, job satisfaction and staff well‐being.
7. Facilitate resolution of clinical practice challenges through supportive and corrective supervision.	Nurse manager competency and a safe supervisory environment are antecedents of effective supervision. Reflection and professional development are processes that support improved performance and quality of care.	Conclusive statement, Theme 2.1.3: Nurse managers encounter difficulties supervising registered nurses with knowledge and skills gaps and acknowledge limitations in their own supervisory knowledge and capacity. Conclusive statement, Theme 2.2.2: Inadequate leadership and quality assurance monitoring weaken accountability and standards of care. Conclusive statement, Theme 2.2.3: Fair and effective conflict management and staff well‐being support, including counselling and wellness programmes, are needed to promote a positive workplace culture and manage work‐related stress.	The findings support a balanced supervisory approach that combines supportive guidance with appropriate corrective action. Nurse managers should identify the causes of practice‐related difficulties, agree on improvement measures, provide necessary support and follow up on corrective actions while maintaining fairness, accountability and professional respect.
8. Establish a structured, continuous clinical supervision process that promotes safe and competent nursing practice.	A safe environment and competent supervision are antecedents of effective clinical supervision. Establishing a relationship, reflection and professional development are core processes. Improved performance, job satisfaction and quality of nursing care are expected outcomes.	Conclusive statement, Theme 2.2.2: Inadequate leadership and quality assurance monitoring weaken accountability and standards of care. Conclusive statement, Theme 2.2.4: Nurse managers need to orient new employees, clarify expectations, strengthen supervisory knowledge and skills and establish clear supervisory structures and roles. Conclusive statement, Theme 2.2.1: Human and material resource shortages and limited access to policies, guidelines and protocols compromise supervision.	The findings directly support formalising supervision through defined responsibilities, scheduled and documented supervisory encounters, clear procedures and systematic follow‐up. Continuity also requires appropriate resources, clear supervisory roles, staff orientation and ongoing development of nurse managers' supervisory competence.

##### 2.6.1.2 Step 1.2 Guideline Development

Scope and purpose of clinical supervision guideline development are concerned with the overall aim of the guideline, the specific health questions and the target population. In this step of guideline development, the scope and purpose of the guidelines were clearly defined. The overall aim of the study was to develop and evaluate clinical supervision guidelines for nurse managers at public hospitals in Lesotho and establish expert consensus on their content. The research question that guided the development of the guidelines was as follows: ‘What clinical supervision guidelines can be developed for nurse managers to support registered nurses during clinical supervision at public hospitals in Lesotho?’ The purpose of the guidelines was to promote the support provided to registered nurses by nurse managers during clinical supervision at public hospitals in Lesotho. The scope of the guidelines was derived from the findings of Phases 1 and 2 of the study.

Stakeholder involvement focused on the extent to which the appropriate stakeholders developed the guidelines and represented the views of their intended users, such as managers and registered nurses. The nurse managers and registered nurses were the key stakeholders in the development of these guidelines. The empirical phase of this study examined the views, preferences and challenges of the target populations, specifically registered nurses and nurse managers. The next domain for guideline development involves the rigour of the development process.

During the development of evidence‐informed and consensus‐based clinical supervision guidelines, study findings from Phases 1 and 2 were systematically incorporated to ensure both rigour and relevance. Empirical data directly informed the structure and content of the guidelines. Key components, including antecedents, processes and outcomes derived from concept analysis, were integrated with the conclusive statements drawn from Phase 2 to formulate the final guidelines. Furthermore, the researcher ensured a clear and explicit link between the guidelines and the supporting evidence (Table 1). Supervisors reviewed the guidelines, and a procedure for updating them was proposed. The panel of experts involved in evaluating the guidelines included national and international qualified experts from private and public hospitals, Ministries of Health, Nursing Services Managers and non‐governmental organisations working in nursing, policy formulation and guideline development. The involvement of all these experts helped the researcher acquire a reliable consensus.

In developing the clinical supervision guidelines, efforts were made to ensure that guidelines were clear and precise. This was achieved by using direct, action‐oriented wording, steering clear of vague expressions and providing clear definitions for essential terms. To address potential ambiguities, pilot testing involved three nurse managers purposively selected from public hospitals in Lesotho. They were selected based on their professional experience and familiarity with nursing management and clinical supervision, enabling them to assess the clarity, relevance, comprehensibility and practical applicability of the proposed clinical supervision guidelines within the public hospital context. The pilot testing was undertaken to identify ambiguities, unclear wording and any aspects of the guidelines that required clarification before finalisation. The nurse managers reviewed the guidelines and provided feedback on their content, wording, interpretation and applicability to routine clinical supervision. Their feedback was examined to identify areas requiring clarification or modification. The relevant sections were subsequently reviewed and refined to improve clarity, consistency and ease of interpretation before the final guidelines were submitted for expert consensus. Furthermore, key recommendations were highlighted using formatting tools, including bullet points, bold font and shaded boxes. These were also compiled into a dedicated section for easy reference, with consistent labels applied throughout.

Applicability addressed potential challenges and enablers related to implementing the clinical supervision guidelines, including strategies to support its adoption and the resources required. To enhance applicability in developing clinical supervision guidelines, the researcher provided actionable guidance and practical tools to aid implementation in real‐world contexts. Consideration was given to both factors that could facilitate uptake and those that might present obstacles, enabling users to anticipate and effectively manage these issues. The guidelines further stressed the resource demands, such as time, staffing and training, associated with successful implementation.

In developing the clinical supervision guidelines, though the study received no external funding, it was explicitly stated that the funder's perspectives had no impact on the guidelines or overall content. Furthermore, all individuals involved in the process, including the researcher, supervisors, nurse managers, experts and registered nurses, provided full disclosure of any potential conflicts of interest. These disclosures were carefully reviewed and appropriately managed to uphold the objectivity, credibility and transparency of the guideline development process.

##### 2.6.1.3 Step 1.3 Expert Evaluation and Refinement

The resulting guidelines were organised into eight guideline statements and subsequently subjected to expert evaluation through a three‐round Electronic Delphi (e‐Delphi) process, using a consensus agreement index ≥ 0.70. This process enabled the proposed guidelines to be reviewed and refined for relevance, clarity and applicability to clinical supervision in public hospitals in Lesotho.

#### 2.6.2 Step 2: Evaluation of Clinical Supervision Guidelines

The clinical supervision guidelines for nurse managers were evaluated to establish expert consensus using a quantitative e‐Delphi method, following the approach described by Romero Collado (2021). The aim of evaluating the clinical supervision guidelines for nurse managers in public hospitals in Lesotho was to establish expert consensus on their content. The objectives of the three rounds were as follows: Round 1 aimed to evaluate the preliminary clinical supervision guidelines and identify necessary revisions. Round 2 aimed to evaluate the revised guidelines and assess the adequacy of revisions made in response to feedback from Round 1. Round 3 aimed to conduct the final evaluation of the guidelines and determine whether the predefined consensus threshold had been achieved. The researcher, with the support from supervisors who are experts in qualitative and quantitative research, coordinated the e‐Delphi process, distributed questionnaires, collated ratings and comments, revised the guidelines and communicated with panellists. Unique identifiers were assigned to participants, consent forms were collected separately from their responses, and the electronic platform was configured to mask IP addresses and disable email tracking. Experts participated remotely and asynchronously, with a 30 days' response period for Round 1 and a one‐week response period for Round 2. The deadline for completing Round 3, which also had a one‐week response period, was communicated by email. Expert evaluation was conducted using a researcher‐developed questionnaire. The questionnaire was designed to assess the proposed clinical supervision guidelines' validity, reliability, applicability, clarity, relevance, comprehensiveness, flexibility and acceptability using a four‐point Likert scale. Experts rated the individual guideline items to determine their level of agreement with the proposed content and quality. The researcher‐developed questionnaire therefore served as the instrument for collecting expert judgements, rather than constituting an administration of the complete AGREE II instrument. Data from the three rounds of the e‐Delphi survey were analysed using descriptive statistics. For each guideline item, the frequencies of expert ratings were calculated. Consensus was defined as at an agreement index of ≥ 0.70. A consensus threshold of an agreement index of ≥ 0.70 among participating experts was established a priori. This threshold was considered acceptable because Delphi methodology does not prescribe a universally standardised definition of consensus and thresholds ranging from ≥ 0.70 to ≥ 0.80 are commonly used in health research (Niederberger and Spranger 2020). For items rated on a four‐point Likert scale, the agreement index was calculated by dividing the number of experts who selected either agree (n_3_) or strongly (n_4_) agree by the total number of experts (N) who participated in the respective e‐Delphi round, yielding a value between 0 and 1. Agreement indices were rounded to two decimal places, where applicable. Items meeting the predefined consensus threshold were retained, while those not meeting the threshold were reviewed and revised, where appropriate, in light of expert feedback before being circulated in subsequent rounds. Responses from each round were compared to assess changes in agreement following revisions. Qualitative comments provided by experts were reviewed thematically to identify suggested amendments, clarify ambiguous wording and improve the validity, reliability, clarity, relevance, applicability, comprehensiveness, feasibility and acceptability of the guidelines. The final round determined whether the revised guideline items met the predefined consensus threshold. The reporting of the expert consensus process was guided by the Accurate Consensus Reporting Document (ACCORD) checklist to promote comprehensive reporting of the e‐Delphi methodology, expert panel characteristics, consensus procedures, findings and modifications made to the clinical supervision guidelines during the successive rounds. The steps are presented in Table 2 below. The experts assessed each guideline as a whole, each specific action and they assessed them for validity, reliability, clarity, relevance, applicability, comprehensiveness, feasibility and acceptability.

**TABLE 2:** e‐Delphi components and descriptions.

e‐Delphi component	Description
Identification of a research problem	The identified problem was the lack of clinical supervision guidelines, resulting in the uncoordinated implementation of clinical supervision in Lesotho, within a context of limited supervisory preparation among emerging nurse managers.
Construction of a draft questionnaire	A 4‐point Likert‐scale questionnaire, piloted with three local experts, was used to evaluate the proposed clinical supervision guidelines. Experts rated the guidelines using a four‐point Likert scale: 1 = strongly disagree, 2 = disagree, 3 = agree and 4 = strongly agree. The assessment criteria included validity, reliability, applicability, clarity, relevance, comprehensiveness, flexibility and acceptability. Experts could recommend changes.
Selection of panel of experts	The panel of experts comprised 21 qualified nurse specialists. Experts were purposively identified based on their expertise and experience in clinical supervision, nursing management, education, research, guideline development and policy.
Questionnaire distribution	Questionnaires were distributed to individual panel members using links to Google Forms and Docs. Panel members were allowed at least 30 days to submit their first‐round responses. They were subsequently invited to complete the questionnaire a second and a third time for the guidelines that did not achieve consensus in the first and second round.
Data analysis and feedback to the panel	Data analysis was guided by the level of consensus reached for each clinical supervision guideline. In this study, consensus (agree to strongly agree) was considered significant at an agreement index of ≥ 0.70. Virtual briefing sessions were held through Google Meet.
Report on findings	Reporting encompassed the number of clinical supervision guidelines assessed in each round, indicating those that were accepted, reassessed, revised and ultimately accepted.

### 2.7 Measures to Ensure Rigour

Throughout the formulation, development and evaluation of the clinical supervision guidelines, the researcher emphasised key quality attributes to ensure their rigour and trustworthiness, including validity, reliability, applicability, relevance, comprehensiveness, acceptability, flexibility, proper documentation and regular review (Mahtani 2015; Brouwers et al. 2010). The guidelines were grounded in empirical findings from Phases 1 and 2 and their validity and reliability were strengthened through expert consensus using the e‐Delphi technique. Their applicability and relevance were ensured by targeting nurse managers in public hospitals in Lesotho and focusing on supporting registered nurses to improve quality of care. Comprehensiveness was achieved by addressing both the structure and processes of clinical supervision, while effectiveness relates to the anticipated improvement in nursing care outcomes following implementation. Acceptability was confirmed by alignment with the vision and mission of the Ministry of Health and Nursing Directorate and endorsement by the expert panel. Flexibility was incorporated by considering the contextual needs and preferences of registered nurses. Meticulous documentation was maintained throughout the study to support transparency and future use. Finally, to ensure continued alignment with emerging evidence, the guidelines will be reviewed and updated every 5 years.

## 3 Results

This section presents clear, actionable clinical supervision guidelines designed to promote support for registered nurses, enabling nurse managers to provide structured, high‐quality clinical supervision that aligns with professional standards and institutional goals. It also describes the process of establishing expert consensus on the guidelines.

### 3.1 Clinical Supervision Guidelines for Nurse Managers

Table 3 below depicts the proposed clinical supervision guidelines for nurse managers in public hospitals in Lesotho.

**TABLE 3:** Proposed clinical supervision guideline.

Guideline	Actions
Guideline 1: Conduct direct clinical supervision to support registered nurses in the safe and effective delivery of patient care.	Conduct regular ward‐based clinical supervision through observation of nursing practice. Observe registered nurses performing clinical procedures and provide immediate guidance where necessary. Review clinical decisions and nursing interventions with registered nurses to promote safe practice. Identify clinical practice gaps during supervision and agree on appropriate improvement measures. Provide opportunities for registered nurses to discuss difficult or complex clinical cases. Use case‐based discussions to strengthen clinical reasoning and decision‐making. Document key supervision activities, findings and agreed follow‐up actions.
Guideline 2: Facilitate clinical learning and professional development through supportive supervision.	Identify individual learning needs from observed clinical practice. Provide bedside teaching, coaching and demonstration of appropriate nursing procedures. Facilitate mentorship and peer‐learning opportunities based on identified clinical needs. Organise targeted learning sessions in response to recurrent clinical practice gaps. Encourage registered nurses to consult current clinical guidelines and evidence when addressing practice problems. Support newly appointed registered nurses through structured clinical orientation and follow‐up supervision. Review progress on identified learning needs during subsequent supervision sessions.
Guideline 3: Promote reflective clinical practice and evidence‐informed decision‐making among registered nurses.	Facilitate reflective discussions following significant, complex or challenging patient‐care situations. Encourage registered nurses to critically examine their clinical decisions and interventions. Discuss current evidence and clinical guidelines relevant to identified practice issues. Use clinical cases, patient outcomes and practice experiences as learning opportunities. Encourage registered nurses to identify alternative approaches to clinical problems. Support registered nurses in translating relevant evidence into everyday clinical practice. Document recurrent clinical issues requiring further learning or follow‐up.
Guideline 4: Provide constructive clinical feedback to strengthen nursing practice and patient safety.	Provide timely, specific and respectful feedback following observation of clinical practice. Reinforce appropriate clinical practices and correct unsafe practices promptly. Discuss the rationale for corrective guidance with the nurse concerned. Agree on practical actions for addressing identified clinical practice gaps. Reassess practice after feedback to determine whether improvement has occurred. Encourage registered nurses to seek feedback and clarification regarding clinical decisions. Maintain appropriate records of significant supervision findings and follow‐up.
Guideline 5: Facilitate clinical problem‐solving and management of complex patient‐care situations.	Encourage registered nurses to present complex clinical cases for discussion during supervision. Guide systematic assessment, clinical reasoning and prioritisation of patient needs. Facilitate discussion of appropriate interventions, referrals and escalation of care. Support registered nurses in recognising and responding to changes in patients' clinical conditions. Promote consultation with relevant healthcare professionals when cases exceed the nurse's scope of practice. Review the management of selected cases to identify lessons for future practice. Follow up on unresolved clinical concerns identified during supervision. Utilise tools such as checklists and performance tracking systems to ensure compliance with care standards. Evaluate patient outcomes and satisfaction to identify areas for improvement regularly.
Guideline 6: Promote supportive interpersonal relationships that enable registered nurses to discuss clinical and professional concerns.	Maintain an approachable and respectful supervisory relationship with registered nurses. Create a safe environment for registered nurses to disclose clinical uncertainties and practice difficulties. Listen actively to registered nurses' concerns and provide appropriate professional guidance. Use coaching and supportive counselling when registered nurses experience difficulties in clinical practice. Facilitate constructive discussion of disagreements arising from clinical practice. Encourage peer support and collaborative learning among registered nurses. Refer concerns requiring specialised psychosocial or professional support through appropriate channels.
Guideline 7: Facilitate resolution of clinical practice challenges through supportive and corrective supervision.	Identify the clinical or professional factors contributing to observed practice problems. Discuss concerns directly with the nurse in a respectful and non‐punitive manner. Distinguish between knowledge or skill deficits, contextual barriers and intentional non‐compliance. Agree on specific corrective or learning actions with the registered nurse concerned. Provide coaching, demonstration or mentorship where the problem reflects a knowledge or skills gap. Reassess the nurse's practice after agreed corrective measures have been implemented. Escalate persistent or serious clinical risks through established professional channels where necessary.
Guideline 8: Establish a structured, continuous clinical supervision process that promotes safe and competent nursing practice.	Develop a clinical supervision schedule based on patient‐care needs and identified practice priorities. Use standardised clinical supervision tools to guide observation, discussion and documentation. Prioritise high‐risk, complex and frequently performed clinical practices for supervision. Ensure continuity of supervision through appropriate delegation of clinical supervision activities to competent senior registered nurses when necessary. Review supervision findings periodically to identify recurring clinical practice issues. Use supervision findings to inform targeted teaching, mentoring and practice improvement. Maintain confidentiality and professional integrity when documenting and discussing supervision findings.

The clinical supervision guidelines were conceptually organised according to their predominant structural function, process function or both to support comprehensive and effective clinical supervision. Guideline 8 was classified as structural, Guidelines 1, 2, 3, 4, 5 and 7 were classified as process‐oriented and Guideline 6 was considered to have both structural and process characteristics. The categories represent the predominant function of each guideline rather than mutually exclusive classifications.

### 3.2 Results of Evaluation of the Clinical Supervision Guidelines

Evaluation of the clinical supervision guidelines was conducted using a quantitative e‐Delphi approach, following the steps described by Romero Collado (2021). The following symbols were used to guide and interpret the evaluation process: *n* represents the number of guidelines assessed; *n* ^f^ denotes the number of final guidelines for which consensus was achieved; ✓ indicates guidelines accepted (agreement index ≥ 0.70); ✗ indicates guidelines removed (disagreement index ≥ 0.70); ± indicates guidelines requiring reassessment (agreement index < 0.70); and + indicates guidelines added by experts. A description of the e‐Delphi process for experts and the outcomes of the clinical supervision guideline evaluation is presented below.

#### 3.2.1 e‐Delphi Experts

The e‐Delphi process was conducted over three iterative rounds to achieve expert consensus on the proposed clinical supervision guidelines. A panel of 21 qualified nursing experts, drawn from national and international governments and non‐governmental organisations specialising in healthcare, human behaviour, research, education and policy development, participated in the evaluation of the draft clinical supervision guidelines. Experts were purposively identified based on their expertise and experience in clinical supervision, nursing management, education, research, guideline development and policy. Their qualifications ranged from nursing multiple‐diplomas to doctoral degrees and their roles included nursing education, clinical practice, nursing management, regulation, quality assurance and policy‐related responsibilities. Their relevant experience ranged from 5–10 years to more than 30 years. The expert panel comprised national and international experts from Lesotho, Kenya, Tanzania, Malawi, Zambia, Namibia, Eswatini and the United States of America. To minimise selection bias, experts were selected using predefined eligibility criteria and representation was sought across different countries and areas of expertise. Initially, a total of 26 experts who met the eligibility criteria were invited to participate in the e‐Delphi evaluation process. Of these, 21 consented and participated in Round 1, giving an initial participation rate of 80.8% (21/26). Twenty of the 21 Round 1 participants completed Round 2, representing a retention rate of 95.2%. The same 20 experts completed Round 3, representing 100% retention from Round 2. Thus, the panel comprised 21 participants in Round 1 and 20 participants in each of Rounds 2 and 3. These figures distinguish initial participation from retention across successive rounds.

#### 3.2.2 Round 1 e‐Delphi Results

In Round 1, all eight proposed guidelines (Guidelines 1–8) were assessed by the expert panel against the eight criteria. Guidelines 1, 2, 7 and 8 achieved the required consensus threshold and were accepted. Their criteria agreement index (CAI) values generally indicated strong expert agreement across the assessment criteria. Guidelines 1 and 8 achieved CAI values of 1.00 for validity, applicability, relevance and acceptability, while Guideline 2 achieved a CAI of 1.00 for reliability, applicability and acceptability. Guidelines 3, 4, 5 and 6 did not achieve the required consensus threshold and were classified as having partial consensus. Their CAI values ranged from 0.57 to 0.67, indicating areas requiring further refinement. The lower scores were particularly evident in clarity, relevance, comprehensiveness and acceptability, although the specific areas varied across the four guidelines. These findings informed the decision to retain Guidelines 3–6 for further review and reassessment in the second e‐Delphi round, while Guidelines 1, 2, 7 and 8 were accepted at this stage. Following Round 1 of the e‐Delphi process, the guidelines were revised to incorporate experts' suggestions for grammatical corrections, improved wording and the removal of ambiguous terms. Table 4 summarises the outcome of the first round of e‐Delphi.

**TABLE 4:** Summary of Round 1 of expert evaluation.

*n*	*n* ^f^	✓	✗	±	+
8	4	4	0	4	0

A detailed description of the summary presented in Table 4, including the agreement index for each clinical supervision guideline in the first round of e‐ Delphi, is given in Table 5.

**TABLE 5:** Detailed outcome of e‐Delphi rounds of expert evaluation.

Guideline	Criteria assessment index								Consensus outcome
	Validity	Reliability	Applicability	Clarity	Relevance	Comprehensiveness	Flexibility	Acceptability	
e‐Delphi round 1									
Guideline 1	1.0	0.86	1.0	0.95	1.0	0.81	0.76	1.0	✓
Guideline 2	0.95	1.0	1.0	0.86	0.71	0.76	0.71	1.0	✓
Guideline 3	0.67	0.67	0.62	0.57	0.67	0.67	0.62	0.57	±
Guideline 4	0.67	0.57	0.62	0.67	0.62	0.57	0.67	0.67	±
Guideline 5	0.57	0.67	0.62	0.57	0.67	0.67	0.62	0.57	±
Guideline 6	0.57	0.67	0.67	0.67	0.57	0.62	0.67	0.62	±
Guideline 7	0.95	1.0	1.0	0.86	0.71	0.76	0.71	0.90	✓
Guideline 8	0.90	0.86	1.0	0.95	1.0	0.81	0.76	1.0	✓
e‐Delphi round 2									
Guideline 3	1.0	0.90	1.0	0.75	0.80	0.75	0.90	1.0	✓
Guideline 4	0.65	0.60	0.60	0.60	0.65	0.60	0.60	0.65	±
Guideline 5	0.80	0.75	0.90	1.0	1.0	0.90	1.0	0.75	✓
Guideline 6	0.65	0.60	0.60	0.65	0.65	0.60	0.60	0.60	±
e‐Delphi round 3									
Guideline 4	1.0	0.90	1.0	0.75	0.80	0.75	0.90	1.0	✓
Guideline 6	0.80	0.75	0.90	1.0	1.0	0.90	1.0	0.75	✓

#### 3.2.3 Round 2 e‐Delphi Results

The second e‐Delphi round focused on Guidelines 3, 4, 5 and 6, which had not achieved the required consensus threshold in the first round. The expert panel reassessed these guidelines following further consideration and refinement. Guidelines 3 and 5 achieved consensus and were accepted, while Guidelines 4 and 6 continued to demonstrate partial consensus and were retained for reassessment in the third round. Guideline 3 demonstrated substantial improvement across all eight assessment criteria, with CAI values ranging from 0.75 for clarity and comprehensiveness to 1.00 for validity, applicability and acceptability. Guideline 5 achieved consensus, with CAI values ranging from 0.75 for reliability and acceptability to 1.00 for clarity, relevance and flexibility. These results indicated improved expert agreement regarding the quality and suitability of the two guidelines. Guidelines 4 and 6 continued to fall below the predetermined consensus threshold. For Guideline 4, CAI values ranged from 0.60 to 0.65, while those for Guideline 6 ranged from 0.60 to 0.65. The findings indicated that further refinement and expert reassessment were necessary before these guidelines could be accepted. Consequently, Guidelines 4 and 6 proceeded to the third e‐Delphi round. Further wording changes and minor modifications were made in response to feedback from Round 2. The second round of e‐Delphi is also outlined in Table 5.

#### 3.2.4 Round 3 e‐Delphi Results

The third e‐Delphi round focused on Guidelines 4 and 6, which had not achieved consensus in the preceding rounds. Following further refinement and reassessment, both guidelines achieved the required consensus threshold and were accepted. Guideline 4 demonstrated improved agreement across all eight criteria, with CAI values ranging from 0.75 for clarity and comprehensiveness to 1.00 for validity, applicability and acceptability. Guideline 6 also showed substantial improvement, achieving CAI values ranging from 0.75 for reliability and acceptability to 1.00 for clarity, relevance and flexibility. All criteria for both guidelines achieved CAI values of at least 0.75, indicating that the expert panel's assessments had improved sufficiently to meet the study's consensus requirements. Following Round 3, the guidelines underwent additional language editing by an English language expert, as recommended by the experts, to improve linguistic clarity and consistency. The third round of e‐Delphi is also outlined in Table 5.

## 4 Discussion

The aim of this study was to develop clinical supervision guidelines for nurse managers supporting registered nurses in public hospitals in Lesotho and to establish expert consensus on the guidelines. A researcher‐developed methodological framework, informed by the AGREE II instrument (2017) and Donabedian's model, guided the development of the clinical supervision guidelines, while evaluation was based on Romero Collado's (2021) steps for conducting e‐Delphi. This section discusses the clinical supervision guidelines, providing a rationale for their development and interpretation by explaining how each guideline addresses identified gaps in clinical supervision practice, supports nurse managers in their supervisory roles and how it potentially contributes to improved professional support for registered nurses and quality of patient care.

### 4.1 Guideline 1

Effective clinical supervision requires an appropriate supervisory structure, including competent nurse managers, adequate time for supervision, defined supervisory responsibilities, appropriate supervision tools and access to clinical guidelines (Yilmaz 2020). These structural conditions are distinct from resource management because the emphasis is not on allocating resources but on creating the conditions necessary for clinical observation, guidance and professional support. Donabedian (2003) conceptualises structure as the attributes of the setting in which care occurs, including human and organisational arrangements. Zonneveld et al. (2024) indicate that effective clinical supervision requires structure, commitment and supportive supervisory relationships. This guideline is strongly supported by the literature, particularly the emphasis placed on clinical supervision as a mechanism for improving the quality and safety of care. The Ministry of Health Nursing Directorate has a mandate to strengthen nursing and midwifery practice and services, promote evidence‐based and evidence‐informed practice, develop competency‐based pre‐service and in‐service education and strengthen registered nurses' capacity in monitoring, evaluation and reporting (Government of Lesotho, Ministry of Health 2021). The Lesotho Nursing Council also establishes standards for nursing practice and a national scope of practice and competency framework against which nursing performance can be understood (Lesotho Nursing Council 2014). Thus, the proposed guideline can function as an operational bridge between national professional standards and the day‐to‐day clinical supervisory responsibilities of nurse managers.

Driscoll et al. (2019) indicate that clinical supervision enables practitioners to develop knowledge and competence, assume responsibility for their practice and enhance the safety of care, particularly in complex situations. The authors also identify clinical supervision as an important organisational mechanism for ensuring quality and safety while supporting continuing professional development. Bifarin and Stonehouse (2017) similarly argue that supervision promotes accountability and contributes to quality care.

Direct observation of clinical practice, discussion of clinical decisions, case review and immediate guidance represent the core processes of clinical supervision (Kusakli 2020). These activities allow nurse managers to identify deviations from expected practice and provide corrective or developmental guidance. This is consistent with evidence that clinical supervision provides opportunities for reflection, critical thinking and feedback (Zonneveld et al. 2024). A systematic review also found evidence that clinical supervision can improve processes of care, which involves direct clinical supervision to support registered nurses in the safe and effective delivery of patient care (Snowdon et al. 2017), although the evidence base remains heterogeneous.

### 4.2 Guideline 2

Guideline 2 guides nurse managers to facilitate clinical learning and professional development through supportive supervision. The structural component of facilitating clinical learning and professional development includes competent nurse managers, access to learning materials, clinical guidelines, mentorship arrangements and opportunities for continuing professional development (Ansah Ofei et al. 2021). These are enabling conditions rather than resource‐management activities when they are specifically directed towards identified clinical learning needs. Vlerick et al. (2024), for example, found that mentoring programmes for specialised and advanced practice nurses commonly incorporated workshops, work shadowing and formal or informal mentoring. Driscoll et al. (2019) identify the formative function of clinical supervision as the continued development of the supervisee's skills, abilities and understanding in clinical practice. Bifarin and Stonehouse (2017) also identify formative supervision as supporting the development of knowledge, skills and attributes associated with problem‐solving and critical‐thinking abilities.

This guideline is consistent with the Nursing Directorate's mandate to develop sustainable competency‐based pre‐service and in‐service education systems and to strengthen nursing and midwifery practice (Government of Lesotho, Ministry of Health 2021). It also complements the Lesotho Nursing Council's competency framework, which defines the competencies expected of registered nurses within the Lesotho healthcare system (Lesotho Nursing Council 2014). Clinical supervision can therefore provide the practice‐level mechanism through which competency expectations are translated into continuous learning, assessment, feedback and professional development. The guideline's emphasis on bedside teaching, coaching, demonstration and mentorship is particularly consistent with clinical supervision. Supervision becomes an educational intervention when the nurse manager identifies a practice gap, provides teaching or coaching and subsequently reassesses the nurse's practice. Evidence from a 2024 systematic review indicates that train‐the‐trainer approaches can support registered nurses' continuing professional development, although implementation and effectiveness vary across contexts (Nexo et al. 2024).

### 4.3 Guideline 3

This guideline promotes reflective clinical practice and evidence‐informed decision‐making among registered nurses. The necessary structures required to promote reflective clinical practice and evidence‐informed decision‐making include access to current clinical guidelines, evidence‐based resources, opportunities for case discussion and nurse managers who possess appropriate clinical and reflective‐practice competencies (Lu et al. 2022; Henneman 2017; Vaismoradi et al. 2015; Sermeus 2016). These conditions support registered nurses' ability to examine their practice critically. Reflection, case discussion, critical examination of clinical decisions and integration of evidence are highly compatible with clinical supervision. This guideline also aligns closely with the strategic direction of the Lesotho Nursing Directorate, which explicitly identifies the promotion of research, evidence‐based and evidence‐informed practice as a strategic objective (Government of Lesotho, Ministry of Health 2021). Consequently, the proposed clinical supervision process provides a practical mechanism for operationalising evidence‐informed practice at ward level.

Valiee and Salehnejad (2020) identified reflection, critical thinking and feedback as important components of effective clinical supervision. The proposed actions of facilitating reflection following challenging clinical situations, critically examining decisions and interventions, discussing clinical cases and considering alternative approaches are therefore highly consistent with the literature. Driscoll et al. (2019) and Rashvand et al. (2017) report that clinical supervision can use case presentations and facilitated group discussion to help practitioners reflect on complex cases and identify learning and actions. Reflection can help practitioners examine the complexity of cases.

### 4.4 Guideline 4

Providing constructive clinical feedback strengthens nursing practice and patient safety. The structural requirements of providing constructive feedback include availability of competent nurse managers, standardised supervision tools, protected opportunities for feedback and an organisational culture in which feedback is viewed as developmental rather than punitive (Ruben 2016). The nurse manager's competence is particularly important because inappropriate or abusive supervision may adversely affect registered nurses and patient safety. Labrague's (2024) systematic review found substantial negative consequences associated with abusive supervision in nursing, including effects on workforce and care‐related outcomes. The emphasis on constructive feedback by this guideline is also compatible with the regulatory environment in Lesotho. The Lesotho Nursing Council identifies nursing practice standards as benchmarks against which actual performance can be assessed and indicates that these standards can be used to identify deficiencies in care and communicate expectations regarding nursing practice (Lesotho Nursing Council 2014). Clinical supervision can therefore provide a supportive process through which nurse managers translate professional standards into feedback on actual practice, rather than relying solely on disciplinary or compliance‐oriented approaches.

Feedback should be timely, specific, respectful and linked to observed clinical practice (Crawford et al. 2017). The process should involve identifying the practice gap, discussing its clinical significance, agreeing on corrective action and subsequently reassessing practice. Evidence from a 2024 systematic review of audit and feedback indicates that registered nurses perceive feedback as useful for identifying gaps between current and best practice and for supporting patient safety (Michl et al. 2024). The proposed actions of providing timely, specific and respectful feedback following observation are therefore consistent with the literature. Feedback should be developmental rather than judgmental because clinical supervision is intended to create a safe environment in which practitioners can critically examine practice. Madula et al. (2018) describe clinical supervision as a supportive relationship that facilitates reflective learning and takes place through a non‐judgmental process.

### 4.5 Guideline 5

Guideline 5 promotes facilitation of clinical problem‐solving and management of complex patient‐care situations. Appropriate structure of clinical problem‐solving and management of complex patient‐care situations requires access to experienced nurse managers, relevant clinical expertise, referral pathways, clinical guidelines and opportunities for multidisciplinary consultation (Mlambo et al. 2021). The nurse manager does not necessarily need to possess expertise in every clinical area but should know when specialist consultation is required. This is particularly relevant to the Lesotho Nursing Council's scope and competency framework, which recognises nursing practice across primary, secondary and tertiary healthcare settings and defines specialist roles within the nursing profession (Lesotho Nursing Council 2014). The supervisory role should therefore include knowing when a clinical issue can be managed through coaching and when it requires referral to a specialist nurse, medical practitioner, or another member of the multidisciplinary team. The core supervisory process involves case presentation, assessment, clinical reasoning, prioritisation, consultation, escalation and subsequent case review (Robertson et al. 2020). This is a particularly strong expression of clinical supervision as it directly connects supervision with actual patient care. Evidence indicates that clinical supervision can improve processes of care, although the strength of evidence varies among professions and clinical contexts (Snowdon et al. 2017). The process is also consistent with WHO's (2022) emphasis on effective, safe, timely and integrated care, because complex cases often require appropriate coordination and escalation across healthcare professionals and levels of care. Evidence also associates formative supervision with problem‐solving and critical‐thinking abilities (Pool et al. 2016; Hegney et al. 2010; Gallagher 2007). Consequently, encouraging registered nurses to present complex cases, systematically analyse clinical problems, prioritise care and consider alternative approaches is strongly compatible with the formative purpose of clinical supervision.

### 4.6 Guideline 6

Guideline 6 provides for the promotion of supportive interpersonal relationships that enable registered nurses to discuss clinical and professional concerns. The structural component of supportive interpersonal relationships is a psychologically safe supervisory environment characterised by accessibility, confidentiality, trust and appropriately trained nurse managers (Sussman and Baldwin 2010). The process involves active listening, supportive discussion, coaching, counselling where appropriate, collaborative problem‐solving and constructive management of disagreements. Such processes distinguish clinical supervision from hierarchical inspection (Apostol et al. 2025). The importance of this relational dimension is reinforced by the distinction between the regulatory and managerial functions of nursing in Lesotho. The Lesotho Nursing Council has responsibility for regulating nursing education and practice and protecting the public through professional standards, whereas the Nursing Directorate is responsible for leading nursing within the health system and strengthening nursing services (Lesotho Nursing Council 2014; Government of Lesotho, Ministry of Health 2021). Within this arrangement, nurse managers are well positioned to provide clinical supervision that helps registered nurses meet professional expectations without reducing clinical supervision to regulatory inspection. Zonneveld et al. (2024) also found that trusting relationships create a safe space for registered nurses to discuss work‐related concerns, reflect and learn. The proposed actions concerning approachability, respect, active listening and creating a safe environment are consistent with the characteristics of an effective nurse manager. Bifarin and Stonehouse (2017) identify relationship‐building, openness, questioning, active listening, focusing and summarising as important supervisory skills.

### 4.7 Guideline 7

Guideline 7 facilitates resolution of clinical practice challenges through supportive and corrective supervision. The structural requirements for resolution of clinical practice challenges include clearly defined professional standards, clinical guidelines, supervisory authority, escalation pathways and appropriately trained nurse managers (Assi et al. 2022). This is preferable to describing the guideline as corrective measures that require conflict resolution, because the latter risks moving the construct towards administrative supervision or disciplinary management (Ozkan Tuncay et al. 2018). The Lesotho Nursing Council's practice standards provide an important external reference point for this process. The Council indicates that nursing practice standards can be used to monitor care, identify deficiencies, communicate expectations, support professional decision‐making and describe incompetent practice (Lesotho Nursing Council 2014). The proposed guideline therefore creates a structured process through which nurse managers can distinguish between a performance concern and a need for developmental support.

The process should begin with identifying the underlying reason for the practice problem. Clinical supervision should distinguish a knowledge or skills deficit from intentional misconduct, contextual barriers or organisational problems (Rubio and Picardo 2017). The appropriate response may therefore be teaching, coaching, demonstration, mentorship or escalation rather than punishment. Chan (2014) emphasises the supportive and non‐judgmental nature of clinical supervision, while De Leo et al. (2023) emphasise the safe and confidential environment in which practitioners reflect on practice. At the same time, the literature does not suggest that clinical supervision should ignore unsafe or incompetent practice. Bifarin and Stonehouse (2017) note that nurse managers should act appropriately where concerns about competence or conduct arise. Similarly, confidentiality may be overridden when unsafe practice or patient risk is identified. Thus, the proposed escalation of persistent or serious clinical risks is justified (Rodríguez‐Martín et al. 2024). This distinction is supported by evidence that supportive supervisory relationships are associated with more positive professional experiences, whereas abusive supervision is associated with adverse workforce and care‐related consequences (Zonneveld et al. 2024; Labrague 2024).

### 4.8 Guideline 8

The eighth guideline is about the establishment of a structured, continuous clinical supervision process that promotes safe and competent nursing practice. Recent evidence specifically identifies structure and organisational commitment as important requirements for effective clinical supervision (Mohamed 2024; Zonneveld et al. 2024). The proposed processes‐scheduled supervision, use of standardised tools, prioritisation of high‐risk practices, documentation, review of findings and follow‐up provide continuity and consistency (Rodríguez‐Martín et al. 2024). The importance of this guideline becomes particularly apparent when considered against the broader history of supportive supervision in Lesotho. Although supportive‐supervision manuals and checklist tools have previously been developed, assessments have identified concerns regarding their dissemination, use, integration and consistency, with supervision described as haphazard and insufficiently coordinated across departments (Government of Lesotho, Ministry of Health 2019). This suggests that the challenge is not simply the absence of supervision but the absence of a sufficiently institutionalised and standardised approach. The present guideline therefore seeks to address this implementation gap by defining how clinical supervision in nursing practice should occur. The guideline is also congruent with the existing institutional responsibilities of the Ministry of Health Nursing Directorate to strengthen nursing practice and services and develop registered nurses' capacity in monitoring, evaluation and reporting (Government of Lesotho, Ministry of Health 2021). At the professional level, it is consistent with the Lesotho Nursing Council's responsibility to set, monitor and maintain standards of nursing education and practice (Lesotho Nursing Council 2014).

Consequently, the proposed guidelines should not be viewed as a parallel supervision system but as a mechanism for operationalising existing national nursing governance and professional standards at hospital and ward level. However, supervision should not become a checklist exercise, standardisation should guide professional judgement rather than replace it (Mohamed and Ahmed 2019). A 2024 systematic review of nursing quality and safety similarly identified communication, adverse events, patient experience, quality improvement and safety culture among important areas for nursing practice (Patrician et al. 2024). Driscoll et al. (2019) describe clinical supervision as a regular process of support and development that should be legitimised and valued by the healthcare organisation. Nantsupawat et al. (2017) further emphasise the importance of agreeing on roles and responsibilities, determining where and how often supervision will occur and establishing organisational support for clinical supervision.

## 5 Conclusion and Recommendations

The clinical supervision guidelines, evaluated and refined through expert consensus, provide a structured, evidence‐informed and consensus‐based framework for strengthening nurse managers' supervision, support and mentorship of registered nurses, while promoting registered nurses' professional competence and well‐being and ultimately enhancing the quality of patient care. It is recommended that the clinical supervision guidelines should be adopted, implemented and evaluated for their potential effectiveness in supporting registered nurses in providing quality nursing care. If effectively implemented and evaluated, these evidence‐informed and consensus‐based guidelines have the potential to enhance registered nurses' competence, motivation and well‐being, ultimately improving patient care quality and ensuring a supportive, efficient and accountable healthcare environment.

## 6 Limitations

The development of the guidelines was informed primarily by concept analysis and qualitative exploration of the experiences and perspectives of registered nurses and nurse managers. While these approaches provided rich, contextually grounded insights into clinical supervision, they may limit the generalisability of the guidelines to other healthcare settings and populations. The findings also reflect participants' subjective experiences and may therefore be influenced by recall, interpretation and contextual factors. The study established expert consensus regarding the content and applicability of the guidelines but did not establish their effectiveness in clinical practice. Consequently, the guidelines should be regarded as evidence‐informed and context‐sensitive, with their applicability strengthened through further empirical testing and evaluation across diverse healthcare settings.

### 6.1 Implications for Nursing Practice

The guidelines emphasise the need to prepare nurse managers through training in clinical leadership, communication, constructive feedback, reflective practice, conflict resolution and performance management. Effective implementation requires protected time, adequate resources, supportive supervision, standardised documentation and clear procedures for escalating patient‐safety concerns. Hospital management should adapt the guidelines to local contexts, secure stakeholder commitment and integrate them into existing nursing policies and quality‐improvement systems. Pilot testing and ongoing evaluation should assess feasibility, acceptability, adherence and effects on registered nurses' competence and well‐being, patient safety and quality of care to inform refinement and wider implementation across Lesotho's public hospitals.

## Author Contributions

**Mpho Zakaria Shelile:** conceptualization, writing – original draft, writing – review and editing, methodology, data curation, formal analysis. **Molekodi Jacob Matsipane:** supervision, writing – review and editing, methodology. **Seepaneng Salaminah Moloko‐Phiri:** supervision, writing – review and editing. **Mmamphamo Miriam Moagi:** supervision, writing – review and editing.

## Funding

The authors have nothing to report.

## Disclosure

Relationship with the broader doctoral research project: This manuscript reports the third phase of a doctoral study focused on the development and evaluation of clinical supervision guidelines for nurse managers in public hospitals in Lesotho. The preceding phases addressed the conceptualisation of clinical supervision and its practice, including the challenges experienced by registered nurses and the support provided by nurse managers. Related findings from these phases have been prepared as separate manuscripts and submitted to other journals. The present manuscript focuses specifically on the methodological process and outcomes of guideline development and evaluation.

## Ethics Statement

This study was approved by the NWU‐Health Research Ethics Committee of the Faculty of Health Sciences of the North‐West University (NWU‐00175‐23‐A1) and the Ministry of Health of Lesotho (ID20‐2024), while informed consent was obtained throughout the study from all participants and respondents. The ethical principles beneficence, respect for human dignity, and justice, as well as considerations of scientific integrity and external review, were adhered to. All participants' anonymity and confidentiality were assured through avoiding association with their identity. Voluntary participation was respected and participants could withdraw from the study at any stage without incurring any consequences whatsoever.

## Consent

Written informed consent was obtained from all participants involved in the study.

## Conflicts of Interest

The authors declare no conflicts of interest.

## Data Availability

The data that support the findings of this study are available on request from the corresponding author. The data are not publicly available due to privacy or ethical restrictions.
